# Supplementary material for: Are carbon emissions trading and green financial instruments synergistic? -Comprehensive quantitative research based on content analysis
Source: PLoS One. 2024 Mar 7;19(3):e0298601. doi: 10.1371/journal.pone.0298601 (PMC10919601; doi:10.1371/journal.pone.0298601)
Supplement: S1 Data — (ZIP) [file pone.0298601.s001.zip › Data/地级市Z_F工作报告.zip!/╜¡╬≈/─╧▓2╩╨/└┤╫╘╣1⁄2╓┌║┼í╛┬φ┐╦╩2╛▌═°í┐_2013.docx]

各位代表：

　　现在，我代表南昌市人民政府，向大会作工作报告，请予审议，并请各位市政协委员和列席会议的同志提出意见。

　　一、2012年工作回顾

　　过去的一年，面对世界经济复苏放缓、国内经济下行压力加大的严峻形势，在省委、省政府和市委的坚强领导下，全市上下齐心协力、攻坚克难，经济社会保持平稳较快发展。

　　一是核心增长极“苗头”初显。省委、省政府和市委、市政府分别召开了动员大会和万人誓师大会，制定出台了一系列政策措施，全市上下打造核心增长极的热情空前高涨，各项工作全面推进，顺利实现了“半年有苗头”的目标。

　　二是进位赶超势头良好。预计全年地区生产总值、财政总收入、社会消费品零售总额分别突破3000亿元、500亿元、1000亿元大关，分别增长约12.5%、21.6%、19%；地方财政一般预算收入240亿元，增长28.3%；500万元以上固定资产投资2620亿元，增长30.8%；规模以上工业增加值945亿元，增长15%左右；实际利用外资26.08亿美元，增长14%；海关出口总额63亿美元，增长11.4%。前三季度，全市社会消费品零售总额、地方财政一般预算收入增幅在全国省会城市中排名第一；固定资产投资、社会消费品零售总额、地方财政一般预算收入增幅连续位居中部省会城市第一；GDP增幅连续位居全省各设区市第一，与2011年相比首位度有所提升。

　　三是增长质量明显提升。税收收入占财政总收入比重达到92%，税收收入占地方财政一般预算收入比重达到83.3％，位居全省和中部省会城市第一；城镇居民人均可支配收入23540元，增长13.5%；农民年人均纯收入9587元，增长13%；万元GDP能耗下降3.6%；居民消费价格总水平涨幅控制在3%左右。实施了总投资1852亿元的60个重大重点项目，集中开工了总投资654亿元的56个重大项目，投资结构得到优化，发展后劲进一步增强。

　　一年来，我们主要做了以下工作：

　　（一）调结构、转方式，产业经济快速发展

　　一是工业经济提速增效。工业项目加快实施。南昌航空城一期、江铃股份30万辆整车等项目进展顺利，欧菲光二期、中节能低碳环保科技园等项目开工建设。全年完成工业投资1100亿元，同比增长30%。主导产业实力增强。汽车、新材料、食品和生物医药三大产业实现主营业务收入1918亿元，增长13.4%；航空制造、纺织服装、电子信息、机电、新能源五大产业规模壮大。园区建设成效明显。南昌经开区经济总量跃升为全省工业园区第二位，小蓝经开区晋升为国家级经济技术开发区，国家小微企业产业发展示范园正式挂牌，昌东、长堎工业园区跻身重点省级工业园区，进贤县开发区进入全省首批筹建省级工业园行列，安义工业园区民用铝型材产能跨入全国三强。二是服务产业更加繁荣。红谷滩万达广场开业，华南城一期加快推进，绿地2.5产业城、南昌国际动漫产业园一期、向塘铁路公路枢纽型物流基地等项目开工建设。全省金融商务区入驻金融机构58家，全省金融产业服务园建设全面启动，华夏银行南昌分行顺利开业，煌上煌等成功上市。滕王阁景区扩建、梅岭景区综合开发、大象湖景区提升改造等项目加快实施，改造提升了34个乡村旅游景点，对48个乡村旅游景点进行了授牌，预计全市旅游接待总人数2537万人次，旅游综合收入202亿元，分别增长21.6%和38.9%。791艺术街区、699文化创意园建成开业，滕王阁古玩城影响扩大。举办各类会展节庆活动130多场。三是现代农业稳步发展。全年粮食总产241.21万吨，实现“九连丰”。新增国家级龙头企业3家、省级龙头企业25家，市级以上龙头企业达到371家。16个大型农业综合开发项目加快实施，“一村一品”强村富民示范村达到605个。农村土地整治和高标准农田建设示范工程扎实推进。南昌县、新建县、进贤县获2012年度全国粮食生产先进县；南昌县被评为国家级现代农业示范区，新建县、进贤县、安义县被评为省级现代农业示范区。

　　（二）抓统筹、强建设，城乡面貌焕然一新

　　一是城乡规划更加科学。国务院正式批复南昌城市总体规划；完成了九龙湖片区规划国际招标和四县城关镇城市总体规划修编工作；在全省率先出台加强农村村民建房规划建设管理实施意见。二是城乡建设大步迈进。重点城建项目进展顺利，洛阳路隧道正式通车；轨道交通一号线全线24个站点开工建设，2号线正式获批；昌南大道东延、生米大桥西延、朝阳大桥、象湖隧道、九洲高架等重点工程加速推进。九龙湖新城起步区建设全面启动。村镇建设加快推进，实施了8个重点示范镇、9个“五位一体”综合示范村和10个秀美乡村建设；完成了768个新农村村点、1686个农村清洁工程点整治，开展了主要通道沿线“五化”综合整治行动，美化房屋6.1万栋，整治道路639.3公里。三是城乡管理力度加大。完成了《南昌市城市管理条例》立法工作，出台了进一步加强城市综合管理的决定。城市管理重点工程顺利实施。文明城市创建、“六治”活动深入开展，“啄木鸟行动”、“小蜜蜂行动”卓有成效。河道采砂专项治理取得重要进展。四是城乡环境更加优化。“森林城乡、花园南昌”和“鄱湖明珠·中国水都”建设加快，乌沙河、象湖景区、抚河截污、幸福渠等水系综合整治工作以及城市排水管网改造有序推进，节能减排深入开展。城市绿化覆盖率达到43%，集中式地表水饮用水源地水质达标率100%，空气质量优良率93.44%，城市环境综合整治定量考核列全省第一。

　　（三）攻难点、活机制，改革开放不断深化

　　一是各项改革深入推进。鄱阳湖生态经济先导区建设步伐加快，机构人员落实到位，总体规划和项目布局加快实施。新建县生米镇成建制划归红谷滩新区管辖。建立了市领导挂点服务重点工业企业制度和人大代表集中视察重点项目、民生工程的机制。成立了市重大重点工程管理办公室，推行政府预选承包商制，建立了建筑市场准入清出机制。出台了扶持县域经济发展20条政策措施。“两县三区综合配套改革试点”和公共资源配套市场化改革进展顺利。城市建设投融资体制改革加快推进。积极开展土地利用“六个试点”，实现全市土地储备“五个统一”。文化体制改革不断深化，广电系统局台分离、两台合并及“三网融合”试点顺利实施。医疗卫生体制改革深入推进，在全国率先实行了医保跨省联网异地结算。二是开放招商成效明显。建立了产业快速决策协调机制。组织参加各类大型招商活动32场，全年签约重大项目114个，投资总额755.31亿元。引进了五十铃、林德气体、航天科工等世界500强和国内500强企业；与中国建筑、中国中铁、上海城建等大型企业签订了战略合作框架协议，与大连万达集团基本达成了投资200亿元兴建文化旅游城的意向。非公经济加快发展，占全市经济比重达到57.3%。

　　（四）惠民生、保稳定，社会建设全面推进

　　一是民生工程全面完成。发放小额贷款6.14亿元扶持就业创业，全市城镇新增就业7.8万人，新增转移农村劳动力4.1万人，被国务院授予“全国创业先进城市”。城镇职工基本养老保险、城乡居民社会养老保险、被征地农民养老保险参保人数分别达到121.3万人、144.2万人、3.3万人；城镇基本医疗保险参保缴费人数161.9万人，新型农村合作医疗统筹基金使用率98%；失业、工伤、生育保险取得新进展。累计发放低保资金4.57亿元，为低收入群众发放价格补贴597万元，城乡低保对象的基本生活得到有效保障；免费救治、济困助学、法律援助等覆盖面扩大。保障性住房建设开工率全省第一，累计保障廉租住房家庭14422户，改造城市棚户区2493套，改造农村危房1470户，国有林场棚户区和国有垦区危旧房改造全部开工建设。扶贫开发深入推进，实施项目226个。免费为农民群众送戏823场。顺利解决17.1万农村人口饮水安全问题。32万用户置换了天然气。城区到县城公交实现全覆盖，市民出行更加便利。二是社会事业协调发展。教育事业加快发展，义务教育网点建设和学校标准化建设扎实推进，城乡公办幼儿园建设全面启动，高中阶段教育基本普及，优质教育资源加速拓展，南昌二中、育新学校等新校区扩容招生。创新能力不断提升，获国家创新基金项目36项、省技术发明奖1项、省科技进步奖15项；新增7家省级工程技术研究中心和9家省级重点实验室。人才队伍更加壮大，引进高层次人才1062名。文化事业日益繁荣，农家书屋实现全覆盖，彭友善美术馆正式开馆；多部文艺作品获中宣部“五个一工程”奖和中国电视金鹰奖。卫生服务能力提升，市洪都中医院红谷滩新院、市第三医院朝阳院区等项目进展顺利，疾病预防控制体系更加健全。成功举办第十五届市运会，“全民健身示范城市”试点工作扎实推进。三是社会管理不断创新。“六五”普法深入开展。社区矫正、安置帮教和警务机制建设扎实推进。信访维稳、治安防控成效明显，圆满完成了党的十八大期间的安保任务。生产安全、交通安全、消防安全、食品药品安全、防汛安全、农产品质量安全以及应急管理形势良好，社会保持和谐稳定。四是政府建设持续推进。公开政府信息8.6万条。提请市人大常委会审议地方性法规2件，制定政府规章3件。审结行政复议案件54件。办结人大代表建议和政协提案482件。实施了第七轮行政审批制度改革，审批时间压缩三分之一以上。深入开展“作风大转变、效能大提速、环境大优化”集中整治活动，干部作风明显改观，机关效能有所提升。

　　与此同时，国防建设、人民防空、国家安全、民族宗教、对台事务、外事侨务等工作继续加强；双拥工作成绩突出，荣获全国双拥模范城“七连冠”。人口计生、统计审计、工商税务、质监安监、参事文史、社科研究、档案史志、地名水文、科普气象、老龄慈善、工会、青少年、妇女儿童、残疾人、防灾减灾、爱国卫生、红十字会、机关事务、消费者权益保护等工作取得喜人成绩。

　　成绩来之不易，经验弥足珍贵。一是必须把打造核心增长极作为第一目标。不动摇、不懈怠、不折腾，不折不扣完成省委、省政府和全市人民交付的历史重任。二是必须把加快发展作为第一要务。以提高经济增长的质量和效益为中心，能快则快、好中求快，促进经济持续健康较快发展。三是必须把改革开放作为第一动力。先行先试、领先领跑，不断增强发展的动力和活力。四是必须把改善民生作为第一责任。努力让全市人民共享改革发展成果，全面激发打造核心增长极的热情和干劲。五是必须把优化环境作为第一保障。努力建设廉洁高效的政务环境、民主公正的法治环境、公平诚信的市场环境、健康向上的人文环境和安全稳定的社会环境。

　　各位代表，2012年各项成绩的取得，离不开省委、省政府和市委的正确领导，离不开市人大、市政协的监督指导，离不开各民主党派、工商联、无党派人士、各人民团体和驻昌单位、人民解放军、武警官兵以及离退休老同志的大力支持，离不开全市广大干部群众的团结一心、奋力拼搏。在此，我谨代表市人民政府，向全市人民，向所有关心、支持南昌发展的同志们、朋友们，表示衷心的感谢并致以崇高的敬意！

　　看到成绩的同时，我们也清醒地认识到存在的问题，主要是：经济首位度不高、产业结构不优、创新能力不强、收入水平不高，资源和环境约束更加突出，加快转变经济发展方式任重道远；体制机制不顺、投资环境不优、干部作风不实等问题仍然比较突出。对这些问题，我们将采取有效措施，认真加以解决。

　　二、2013年工作安排

　　2013年是全面深入贯彻落实党的十八大精神的开局之年，也是全面打造核心增长极的关键之年。综合分析国内外发展大势，我们仍处于可以大有作为的重要战略机遇期，同时也面临不少风险和挑战。我们将始终保持清醒的头脑，进一步增强危机感、紧迫感、使命感、责任感，全力以赴抓好今年各项工作。

　　今年政府工作的总体要求是：全面贯彻落实党的十八大精神，高举中国特色社会主义伟大旗帜，进一步解放思想、改革开放、务实奋进，坚持不懈推进大投入、大建设、大发展，坚定不移实施聚焦战略，全面打造核心增长极，为建设富裕和谐秀美江西、全面建成小康社会作出应有贡献。

　　今年经济社会发展的主要预期目标是：

　　地区生产总值增长12%以上；

　　财政总收入增长15.5%；

　　地方财政一般预算收入增长15.5%；

　　全社会固定资产投资增长23%；

　　规模以上工业增加值增长14.5%；

　　社会消费品零售总额增长16%；

　　实际利用外资增长10%；

　　城镇居民人均可支配收入增长12%；

　　农村居民人均可支配收入增长12%；

　　万元生产总值能耗下降3.5%；

　　主要污染物排放总量下降1.5%；

　　人口自然增长率控制在8‰以内；

　　居民消费价格总水平涨幅控制在4%以内。

　　为完成上述目标任务，我们将重点抓好以下五项工作。

　　（一）突出产业发展，推动经济实力再上新台阶。坚持“二三并举”、“双轮驱动”，不断做大总量、优化结构，以产业经济的强劲增长支撑全市经济总量的快速提升。一是强化工业主导地位。持续加大工业投入，坚定不移聚焦工业、做大工业、做强工业。深入实施“双千双百”工程，加快打造三大千亿产业和五个超500亿产业，切实提高五大战略性新兴产业创新发展能力。不断提升园区发展水平，加快建设高新区、南昌经开区、小蓝经开区、桑海开发区等国家级开发区和省级产业园区，着力打造国家小微企业产业发展示范园。突出抓好项目建设和企业培育，重点实施南昌航空城、江铃五十铃整车和20万台发动机、欧菲光二期等40个重大工业项目；加速培育20家前景好、带动力强、贡献大的龙头企业。二是提升服务业发展水平。商贸物流方面，大力推进南昌国际博览中心、南昌之星城市综合体、南昌龙头岗综合码头、向塘铁路公路枢纽型物流基地等重大项目建设；着力建设中央商务区、八一广场中山路商业中心、城东临江商务区等商圈，改造提升洪城大市场，打造商业特色街，挖掘盘活老字号。现代金融方面，加快建设全省金融商务区和金融产业服务园，大力发展新型金融机构和金融组织；加大企业上市力度，力争新增2－3家上市公司。旅游休闲方面，抓好万达文化旅游城、滕王阁景区扩建、小平小道景区扩建、梅岭景区综合开发、鄱阳湖南矶湿地生态旅游区等重点旅游项目建设，抓好旅游集散中心省市共建；加快发展乡村旅游，提升南昌县、安义县乡村旅游水平，加快建设溪霞休闲农业公园和军山湖品蟹休闲旅游区。服务外包方面，加快建设国家服务外包示范城市，努力打造中部地区的服务外包中心。文化产业方面，继续推进南昌国际动漫产业园、文港文化产业园等重点项目建设，进一步提升文化创意产业园区水平，努力建设全省文化产业发展高地。三是巩固农业基础地位。全面落实强农惠农富农政策，充分保护和调动农民生产经营积极性。加大农田水利基础设施投入，全面完成农村土地整治示范工程和高标准农田建设，提升农业综合生产能力，切实保障主要农产品安全有效供给。全力抓好百个重点都市现代农业项目和“七大百亿特色产业”，培育壮大一批龙头企业，进一步提升农业产业化水平。加快推进休闲农业、大型农业综合开发和特色基地建设。

　　（二）突出统筹发展，推动城乡一体迈出新步伐。把城镇化作为扩大内需的主要潜力，继续加大城建投入，着力扩大城乡消费，把生态文明建设放在更加突出的位置，切实提高城镇化质量，促进城乡共同繁荣，力争成为全省统筹城乡经济社会发展一体化的先行区和示范点。一是强力推进城乡建设。加快编制和完善九龙湖新城、朝阳新城、国家小微企业产业发展示范园等区域控制性详细规划；认真编制重点地区、重要地段的特色规划和专项规划；启动南昌大都市区规划编制。集中资源建设红谷滩新区，加快推进九龙湖核心起步区的市政基础设施和公共服务配套设施建设，争创国家绿色生态示范区和智慧新城；加大东湖区、西湖区、青云谱区、青山湖区旧城改造力度，强力推进青山湖西岸开发；深入推进“五大组团”建设。加快实施轨道交通、朝阳大桥、“十横十纵”快速路网、象湖隧道、朝阳新城等城市建设重点项目。加快推进城乡电网建设和城市燃气“一张网”建设。持续开展建筑质量安全执法大检查，加大违法处罚力度。继续推进8个重点示范镇、11个特色小镇和10个秀美乡村、9个“五位一体”综合示范村以及630个左右新农村村点整治建设；着力创建中国秀美乡村·安义；基本完成25条主要通道沿线“五化”综合整治；加快推进105、320国道一级公路建设，大力推动农村公路升级改造和危桥改造。二是突出加强城乡管理。深化城市管理体制机制改革，加快建设街办（镇）城市管理工作办公室和社区城市管理工作站。推动城市管理重心下移及市场化进程，逐步推行“购买环境”、“购买服务”。全力实施城市管理重大项目建设，提升城市道路、桥涵、排水管网、公厕、垃圾站等市政设施改造管养水平和机械化清扫率。开展城市管理年活动，推进“啄木鸟”行动，大力开展市容环境和交通秩序重点整治，突出抓好占道经营、建筑垃圾、违法建设和“三乱”行为专项整治行动。继续抓好农村清洁工程和河道采砂专项整治。切实加强农村村民建房规划建设管理。继续推进全国文明城市创建工作。三是加快建设生态文明。全面落实《低碳城市发展规划》，建设国家低碳试点城市。继续推进“森林城乡、花园南昌”建设，大力实施造林绿化“五大工程”，着力创建国家生态园林城市、国家森林城市。加快打造“鄱湖明珠·中国水都”，全面推进九龙湖、幸福渠、大象湖等水系综合整治，精心建设水环境、水景观和水文化；加快实施污水配套管网、雨污分流、截污工程以及城镇污水处理厂提标改造建设；做好56座病险水库水闸的除险加固，推进鄱阳湖二期第6个单项工程建设，提高城市防洪排涝能力。实行最严格的耕地保护制度、水资源管理制度和环境保护制度，大力开展大气、水环境、重金属和危险废物污染专项整治，建立机动车淘汰倒逼机制，全面完成主要污染物年度减排任务。四是着力扩大城乡消费。积极探索增强消费能力、培育消费热点、优化消费环境的新途径。继续实施“万村千乡”市场工程，加快农产品现代流通体系建设。抓好全国中小商贸流通企业服务体系建设试点，着力抓好集贸市场升级改造、“放心粮店”、“网上粮城”和农副产品平价商店建设。加大对文化、旅游休闲、体育健身、家政、养老服务的政策扶持力度。严厉打击损害消费者权益的行为。

　　（三）突出攻坚克难，推动改革开放取得新突破。用足用好用活省委、省政府赋予的改革权、试验权，不断深化改革开放，全面实施创新驱动，增强民营经济活力，把先行先试的政策优势迅速转化为打造核心增长极的巨大动力。一是加快建设鄱阳湖生态经济先导区。按照“一轴六区”的功能布局，加快编制先导区“三规合一”的发展规划和三年行动计划。进一步明确先导区的重点产业抓手，高新区重点抓好航空产业，南昌经开区和新建县重点抓好国家小微企业产业发展示范园，小蓝经开区重点抓好汽车产业，红谷滩新区重点抓好全省金融商务区和总部经济，桑海开发区重点抓好食品和生物医药产业。认真谋划和推进五十铃商用车、统一食品、正星光电等一批重大项目。科学制定先导区改革创新的路线图和时间表，把先导区打造成为江西“不是特区的特区”。二是不断创新体制机制。继续深化国有企业改革，推进国有资产管理体制创新。大力推进投融资体制改革，做强做大做实投融资平台。全面实施创新驱动战略，着力构建以企业为主体、市场为导向、产学研相结合的技术创新体系。加快户籍制度改革，有序推进农业转移人口市民化，引导农村人口向城市有序转移、周边人口向南昌集聚。深化土地制度改革，推进土地利用“六个试点”，改革征地制度，全面完成土地收储任务，切实提高土地节约集约利用水平。深化行政审批制度改革，全面下放审批权、上收否决权，保留规划、环保监督权，大力开展中介机构专项清理整顿行动，在更宽领域、更深层次上简政放权，进一步优化投资发展环境、释放县区发展潜能。三是着力提升开放招商水平。牢牢把握招商引资“生命线”，实行更加积极主动的开放战略。继续巩固港澳台等传统招商区域，积极开拓东南亚、欧美等新兴市场，加大与长江中游城市群、泛珠三角区域的合作与交流。组织参加赣台会、中博会、泛珠会、香港活动周等招商活动，大力开展有针对性的敲门式招商。突出抓好“招大引强”，力争引进一批世界500强和国内500强企业。加大对机电、高新技术产品出口的扶持力度。鼓励有条件的企业“走出去”。积极申报南昌综合保税区，降低海铁联运成本，做大做强南昌出口加工区、南昌保税物流中心。四是切实增强民营经济活力。以加快民营经济发展作为新一轮开放的突破口和着力点，制定完善支持民营经济发展的政策措施，努力营造尊重创造、全民创业的浓厚氛围，着力解决困扰民营企业发展的突出问题。以县域经济作为民营经济的大舞台，支持南昌县发展汽车及零部件、建筑等重点产业，支持新建县发展汽车装备、出版印刷等重点产业，支持进贤县发展医疗器械、生物医药等重点产业，支持安义县打造华东地区最大建材产业基地，支持湾里区创建国家级旅游度假区。

　　（四）突出民生优先，推动社会建设实现新跨越。坚持把人民利益放在第一位，加快健全基本公共服务体系，加强和创新社会管理，确保社会和谐稳定。一是大力实施民生工程。实施就业优先战略和更加积极的就业政策，全年新增城镇就业6.87万人，新增转移农村劳动力4.11万人, “零就业家庭”就业安置率达到100%。千方百计增加居民收入，及时启动联动机制为低收入群体发放价格补贴。统筹推进城乡保障体系建设，扩大社会保险覆盖面，适当提高参保人员保障水平，加快推进市人力资源和社会保障公共服务中心建设，继续完善社会救助体系、社会福利制度，加快发展慈善事业。全面落实房地产市场调控政策，大力实施“百姓安居工程”，加快建设保障性住房6000套，完善配套设施；加快推进农村、国有垦区和林场的危房改造。加快建设健康城市，重点推进妇女儿童活动中心、青少年宫、残疾人康复中心、老年福利院、全民健身站点等建设；加强重大疾病防控体系建设，大力实施免费健康服务，不断提高市民健康水平。整体推进100个重点扶贫村的扶贫开发。再解决10万农村人口饮水安全问题。大力实施“村邮户箱”工程。二是全面推进社会事业。优先发展教育。合理配置教育资源，维护教育公平，抓好中小学教育网点规划调整，加快九龙湖起步区优质学校建设工程，启动东湖区滨江学校等5所城区九年一贯制学校建设，推进全市中小学生综合实践基地建设，完成市第一中学朝阳校区、实验中学高新校区等优质高中建设，新建、加固和改造中小学校舍面积40万平方米；加快城乡公办幼儿园和普惠性幼儿园建设；加快国家级中等职业教育改革发展示范校建设。强化科技创新。加大重大科技产业化专项资金支持力度，加快推进半导体照明产业基地、航空制造产业基地、生物高技术产业基地等国家级高技术产业基地建设，加快建设瑶湖生态科技城、欧菲光电子信息产业基地，积极申报国家科技金融试点城市。强化人才支撑。创新引才用才机制，使人才成为南昌创新发展的核心优势。繁荣文化事业。加快推进南昌瓷板画研究中心、八大山人真迹陈列馆扩容项目建设，加强县区“三馆一站”等基层文化设施建设，鼓励创作具有南昌特色的文艺精品，大力支持社区文化建设。着力抓好卫生服务。改革支付方式，破除“以药补医”机制；推进县级公立医院改革，稳定乡村医生队伍，提高社区卫生服务水平；加快建设市三医院朝阳院区和市九医院、市皮肤病医院项目。大力开展爱国卫生运动，巩固和发展国家卫生城市成果。加快农产品质量安全体系建设，加强食品药品安全监管，保障人民群众饮食和用药安全。积极发展公共体育事业，深入推进“全民健身示范城市”试点。夯实人口计生基层基础，严格落实人口计生政策。三是不断创新社会管理。充分发挥群众参与社会管理的基础作用，继续深化基层民主建设，增强社区服务功能，积极探索“以人为本，网格化管理、信息化支撑、全程化服务”的社会管理新模式。深入开展法治南昌创建活动，不断推进社区矫正、安置帮教工作。创新信访工作体制机制，依法依规化解各类信访问题。建立健全人民调解、行政调解、司法调解联动的工作体系，完善医患纠纷第三方调解机制。加快治安大防控体系建设，深入开展“打四黑除四害”等社会治安专项整治行动，全力侦破重案、要案。不断完善公共安全体系，大力开展安全生产、消防、交通、地质灾害、食品药品、建筑施工、防震减灾等领域的事故隐患大排查、大整治，不断完善应急预案，加强应急避难场地建设和应急演练，坚决遏制各类重特大安全事故和群体性事件的发生。

　　（五）突出作风建设，推动环境优化开创新局面。进一步加强政府自身建设，全面贯彻落实关于改进工作作风、密切联系群众的八项规定。一是增强依法行政能力。推进科学立法、严格执法，努力做到政府职权法授、程序法定、行为法限、责任法究。坚持科学决策、民主决策、依法决策，凡涉及群众切身利益的决策都充分听取群众意见，凡是损害群众利益的决策都坚决防止和纠正。推进政务公开，加快电子政务建设，充分保障人民群众的知情权、参与权、监督权。切实加大监督力度，健全质询、问责、经济责任审计等制度；自觉接受市人大及其常委会的法律监督、工作监督和市政协的民主监督。及时办理人大代表建议和政协提案，不断提高办结率和满意率。二是切实改进工作作风。坚决反对形式主义、官僚主义。进一步精简“文山会海”，切实改进会风，严格控制全市性会议和重大活动的举办，提倡开短会，说短话；切实改进文风，精简文件简报。认真执行行政问责制度，严肃查处行政不作为、慢作为和乱作为现象。大力开展纠风工作，依纪依法从严查处“吃拿卡要”、强买强卖、执法不公等严重损害投资环境的人和事。三是带头密切联系群众。深入基层调查研究，倾听群众呼声，关心群众冷暖，多办顺民意、解民忧、惠民生的好事实事。坚持向群众学习、向实践学习，经常问政于民、问需于民、问计于民，始终保持与人民群众的血肉联系。四是强化反腐倡廉建设。认真落实党风廉政建设责任制，严格遵守廉洁从政有关规定，建立健全权力运行制约和监督体系，坚决防止、严肃惩处权力寻租、权钱交易等违法违规行为。坚持厉行节约，反对铺张浪费，严格执行住房、车辆配备有关工作和生活待遇的规定。加大腐败案件查处力度，始终保持惩治腐败的高压态势，大力营造干部清正、政府清廉、政治清明的社会环境。

　　各位代表，站在新的发展起点上，我们深感责任重大、使命光荣。让我们紧密团结在以习近平同志为总书记的党中央周围，在省委、省政府和市委的坚强领导下，攻坚克难，开拓创新，全面加快打造核心增长极进程，为促进全市经济持续健康较快发展和社会全面进步作出新的更大贡献！
